# Targeting MYH9 represses USP14-mediated NAP1L1 deubiquitination and cell proliferation in glioma

**DOI:** 10.1186/s12935-023-03050-1

**Published:** 2023-09-28

**Authors:** Zigui Chen, Xin Yan, Changfeng Miao, Longyang Liu, Su Liu, Ying Xia, Weiyi Fang, Dandan Zheng, Qisheng Luo

**Affiliations:** 1 Cancer Center, Integrated Hospital of Traditional Chinese Medicine, Southern Medical University, 13 Shiliugang ST, Guangzhou, 510315 China; 2 Department of Neurosurgery, Affiliated Haikou Hospital of Xiangya Medical School, Central South University, Haikou, 570208 China; 3 Department of neurosurgery, Affiliated Hospital of Youjiang Medical University for Nationalities, Baise, Guangxi 53300 China; 4 Department of Laboratory Medicine, Neurosurgery Second Branche, Hunan Provincial People ’ s Hospital, The First affiliated Hospital of Hunan Normal University), Changsha, Hunan 410005 China; 5 Department of encephalopathy, Liuyang Hospital of Traditional Chinese Medicine, Liuyang, Hunan 410300 China; 6 Department of Radiation Oncology, The First Affiliated Hospital Zhejiang University, Hangzhou, 310009 China

**Keywords:** MYH9, NAP1L1, Glioma, Proliferation, Chemoresistance, Ubiquitination

## Abstract

Myosin heavy chain 9 (MYH9) plays an important role in a number of diseases. Nevertheless, the function of MYH9 in glioma is unclear. The present research aimed to investigate the role of MYH9 in glioma and determine whether MYH9 is involved in the temozolomide chemoresistance of glioma cells. Our results showed that MYH9 increased the proliferation and temozolomide resistance of glioma cells. The mechanistic experiments showed that the binding of MYH9 to NAP1L1, a potential promoter of tumor proliferation, inhibited the ubiquitination and degradation of NAP1L1 by recruiting USP14. Upregulation of NAP1L1 increased its binding with c-Myc and activated c-Myc, which induced the expression of CCND1/CDK4, promoting glioma cell temozolomide resistance and proliferation. Additionally, we found that MYH9 upregulation was strongly related to patient survival and is therefore a negative factor for patients with glioma. Altogether, our results show that MYH9 plays a role in glioma progression by regulating NAP1L1 deubiquitination. Thus, targeting MYH9 is a potential therapeutic strategy for the clinical treatment of glioma in the future.

**Supplementary Information:**

The online version contains supplementary material available at 10.1186/s12935-023-03050-1.

## Introduction

Glioma is a primary malignant tumor most commonly found in the brain. Chemotherapy is one of the more common approaches for glioma treatment because it may be used before or after surgery and is appropriate for people who are not eligible for surgery [1]. Since the high proliferative capacity and chemoresistance of glioma cells limits the effectiveness of conventional therapies, the mortality rate of glioma has been consistently increasing, and the 5-year survival prognosis for patients with glioma remains low. While the mechanisms of drug resistance at the molecular, cellular, and pathological levels in glioma have been extensively investigated, drug resistance continues to pose a major challenge to the treatment of patients with glioma [2, 3]. Therefore, it is extremely urgent to identify biomarkers and drug targets that are effective in improving the prognosis of patients with glioma.

Myosin heavy chain 9 (MYH9), a member of the myosin family of proteins, participates in cell adhesion, polarity, motility, and migration [4]. MYH9 is a skeletal protein that plays a critical role in many human diseases [5–9]. Over the past several years, an increasing number of studies have shown that MYH9 is upregulated in many cancers, such as non-small cell lung cancer [10], colorectal cancer [11], prostate cancer [12], diffuse large B-cell lymphoma [13], renal cell carcinoma [14], glioma [15], and hepatocellular carcinoma [16]. It is a potential tumor promoter that has been reported to enhance tumor proliferation, invasion and migration. Previous research has demonstrated that MYH9-promoted CTNNB1 transcription results in anoikis resistance in gastric cancer [17]. A further study showed that MYH9 overexpression leads to epithelial-to-mesenchymal transition and stemness in hepatocellular cancer [16]. Recently, it was confirmed that overexpression of MYH9 could downregulate tumor suppressor genes to induce the recurrence of pancreatic cancer [18]. In contrast, MYH9 was identified as a tumor suppressor in squamous cell cancers of the head and neck [19]. Nevertheless, the function of MYH9 in glioma is unclear.

In the present research, the molecular function of MYH9 in glioma cells was investigated. MYH9 bound to NAP1L1 and inhibited the ubiquitination and degradation of NAP1L1 by recruiting USP14. The upregulation of NAP1L1 increased its binding with c-Myc and thereby activated c-Myc, inducing the expression of CCND1/CDK4 and thereby promoting glioma cell temozolomide resistance and proliferation. The findings of the present research suggest that MYH9 is involved in the pathogenesis of glioma and is a potential target for its clinical treatment.

## Results

### Upregulation of MYH9 was associated with poor prognosis in patients with glioma

**Fig. 1:**
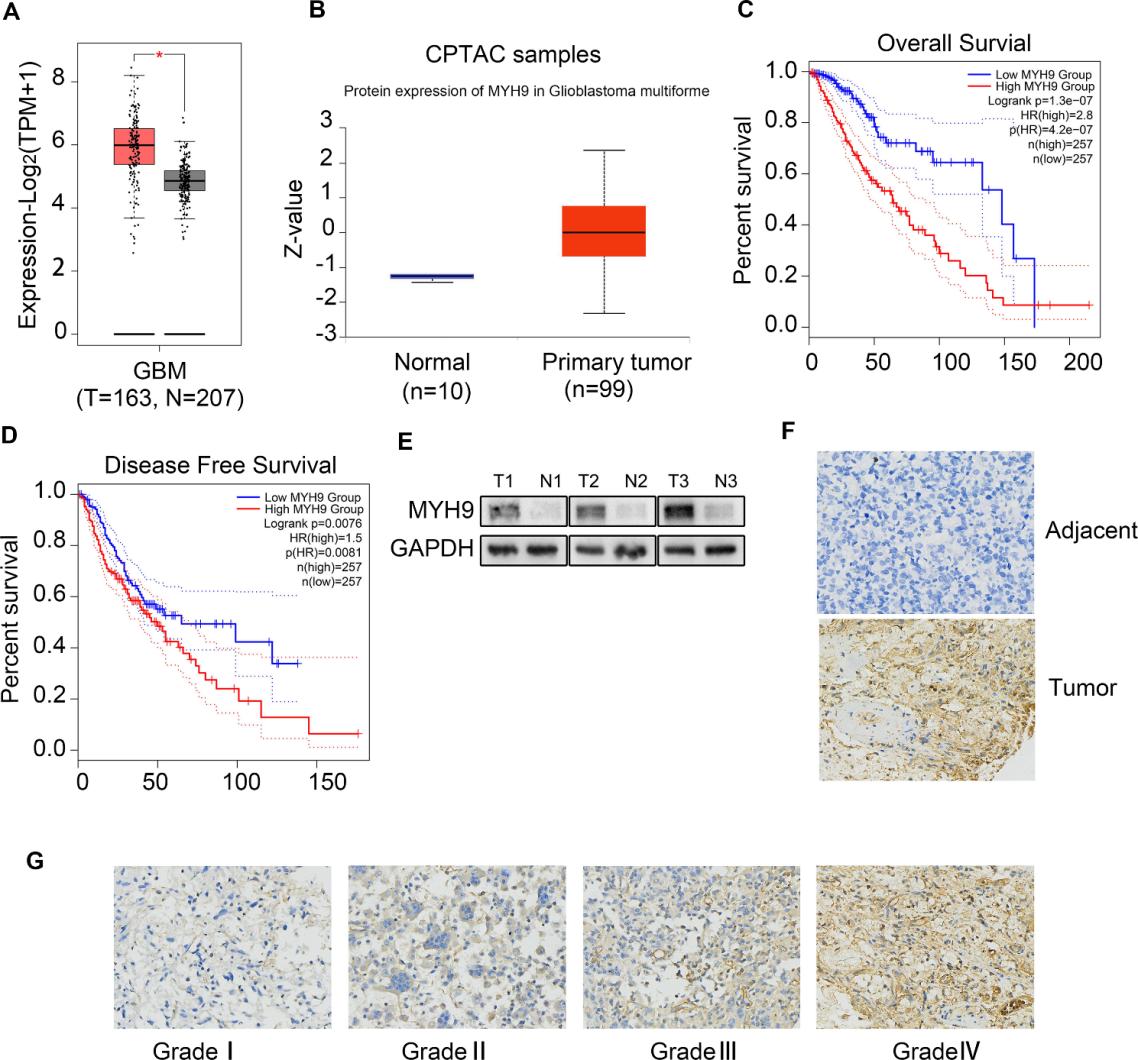
MYH9 is increased in glioma and correlates with poor prognosis of glioma. (A) MYH9 mRNA expression in glioma tissues and para-tumor tissues among the glioma patients obtained from the TCGA database. (B) MYH9 protein level in glioma tissues and para-tumor tissues among the glioma patients obtained from the CPTAC database. (C, D) Kaplan–Meier survival analysis for overall survival (C) and Disease Free survival (D) based on the MYH9 expression data. (E) Western blot of MYH9 protein level in 3 glioma tissues and 3 para-tumor tissues. (F) The expression of MYH9 in tumor and adjacent samples were monitored by immunohistochemistry. (G) Representative images of MYH9 staining in (grade I-IV) glioma tissues (scale bar: 50 μm). Data are presented as the mean ± SD for three independent experiments. *P < 0.05, **P < 0.01, ***P < 0.001

To determine the possible role of MYH9 in glioma, the transcriptional level of MYH9 in glioma tissue was compared with that in normal tissues using GEPIA. We found that MYH9 mRNA expression was significantly upregulated in patients with glioma in the TCGA/GTEx dataset (Fig. 1A). Using the CPTAC portal, we found that the protein expression of MYH9 was upregulated in glioma patients (Fig. 1B). The results of the OS and DFS analyses indicated that the upregulation of MYH9 was negatively correlated with the overall survival of patients with glioma (Fig. 1C, D). The western blotting (WB) results showed higher MYH9 expression levels in three fresh glioma tissues than in three adjacent normal tissues (Fig. 1E). To further determine the expression of MYH9 in glioma tissues, a tissue microarray (TMA) containing 168 glioma tissue samples and 15 peritumoral tissue samples was subjected to immunohistochemical (IHC) staining for MYH9 expression (Fig. 1F). The expression of MYH9 in glioma tissue was also found to be significantly correlated with the pathological grade of the tumor (WHO II vs. WHO III vs. WHO IV) (Fig. 1G). In summary, these findings showed that MYH9 expression in glioma tissues was higher than that in adjacent tissues and that MYH9 acts as an oncogene that can predict poor prognosis in patients with glioma.

### MYH9 enhanced glioma cell proliferation and resistance to temozolomide

**Fig. 2:**
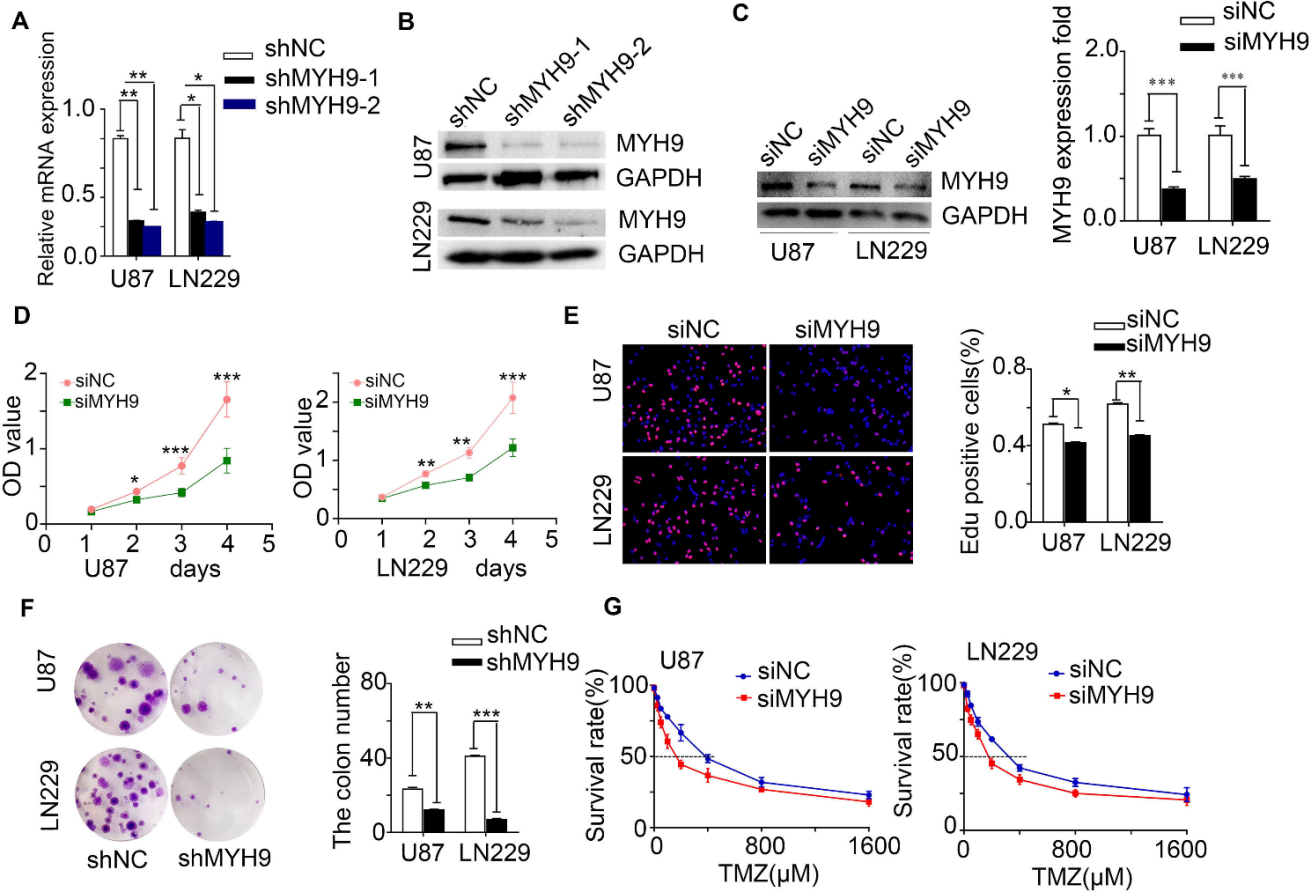
MYH9 enhances cell proliferation and resistance to temozolomide. (A, B) MYH9 expression was measured by RT-PCR assays and western blot in U87 and LN229 cells transfected with shNC ,shMYH9-1 and shMYH9-2. GAPDH was used as a loading control. (C) MYH9 protein level was measured by western blot in U87 and LN229 cells transfected with siNC or siMYH9. GAPDH was used as a loading control. CCK8 assay (D), EdU incorporation assay (E), clone formation assay (F) after MYH9 knockdown. (G) Dose-response curves of U87 and LN229 treated with siMYH9 and siNC respectively following treatment with temazolamide for 48 h. Data are presented as the mean ± SD for three independent experiments. *P < 0.05, **P < 0.01, ***P < 0.001

To explore the role of MYH9 in glioma, glioma cells were transfected using a lentivirus carrying the MYH9-shRNA vector (shMYH9) and a lentivirus carrying a negative control vector (shNC). MYH9 expression was then assessed using qRT‒PCR analysis (Fig. 2A) and western blotting (WB) (Fig. 2B). We found that shMYH9-2 had the highest knockdown efficiency, and we also verified the knockdown efficiency of si-2 siRNA (small interfering RNA-2) (Fig. 2C). The CCK8 assay demonstrated that MYH9 knockdown obviously suppressed the proliferation of U87 and LN229 cells (Fig. 2D). Moreover, MYH9 overexpression significantly promoted glioma cell proliferation. In addition, the EdU assay (Fig. 2E) and clone formation assay (Fig. 2F) confirmed that downregulation of MYH9 decreased the viability of glioma cells.

**Fig. 3:**
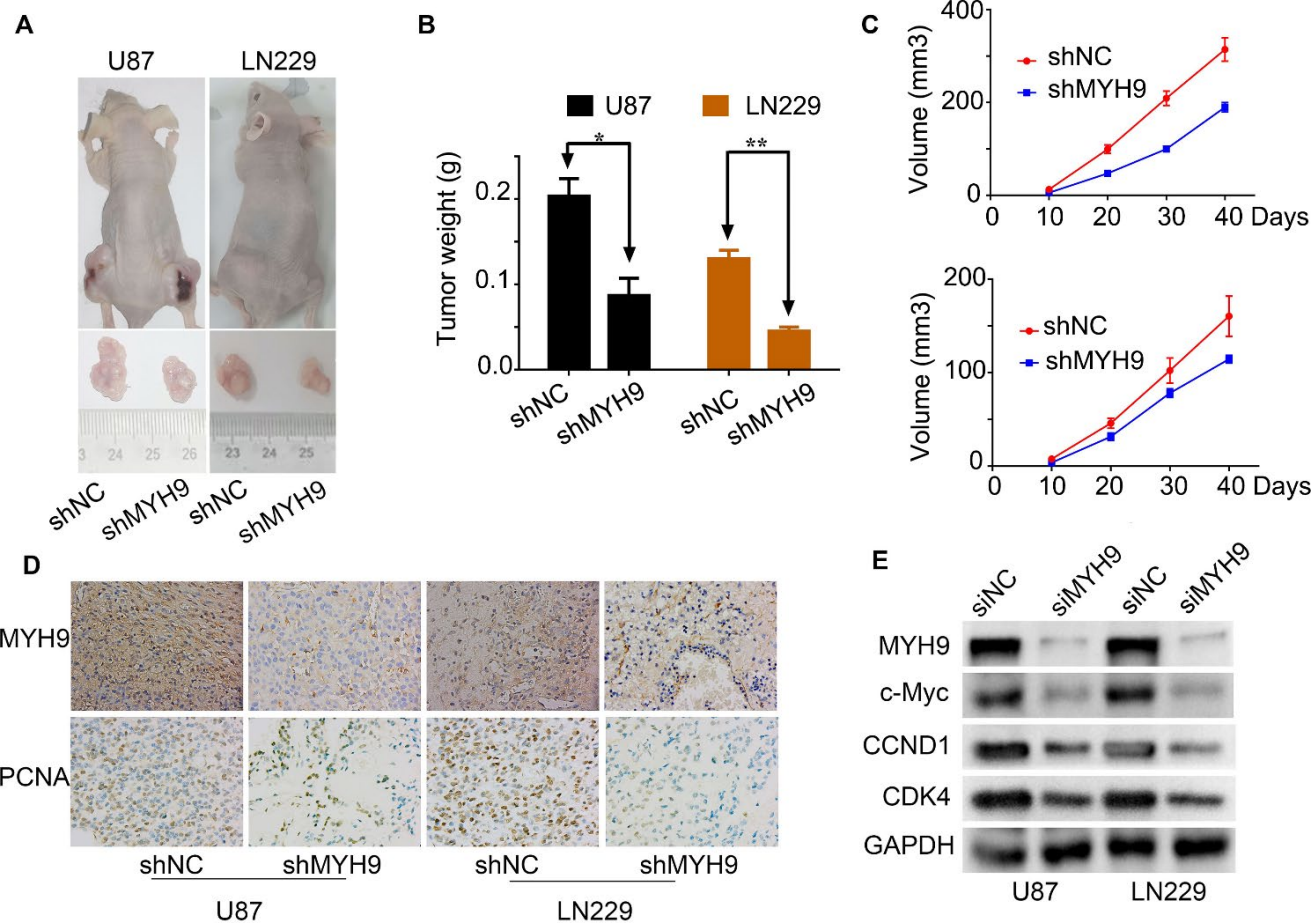
MYH9 increases glioma proliferation and resistance to temozolomide via the c-Myc signaling pathway. Gross morphology of tumors (A) and tumor weight statistics (B) from the indicated groups (n = 5 per group). (C) Tumor volume statistics for each mouse group (n = 5 per group). (D) MYH9 and PCNA was evaluated by immunohistochemical staining. Compared with shMYH9 cells, the shNC cell tumor tissues were high expression. (E) Western blotting analysis of the protein levels of MYH9, c-Myc, CCND1 and CDK4 after transfecting siNC or siMYH9 into U87 and LN229 cells. Data are presented as the mean ± SD for three independent experiments. *P < 0.05, **P < 0.01, ***P < 0.001

In addition, U87 and LN229 cells containing shMYH9 showed enhanced sensitivity to TMZ-induced cell death. Glioma cells were treated with different TMZ concentrations after 48 h, and the inhibition of cell viability was calculated after MYH9 knockdown. The TMZ dose–response curves showed that the IC50 value was 401.39 µM in the control (shNC) group, but it diminished dramatically to 183.00 µM in U87 cells with MYH9 knockdown (Fig. 2G, left); similarly, the IC50 value was reduced from 505.20 µM to 250.05 µM in LN229 cells with MYH9 knockdown (Fig. 2G, right). Interestingly, WB showed that the expression of γ-H2AX, a biomarker for DNA double-strand breaks, was upregulated in glioma cells with MYH9 knockdown (Supplemental Fig. 1A). Moreover, a subcutaneous transplantation animal model was constructed in nude mice using shMYH9-transfected glioma cells. We observed that the shMYH9 group of mice had smaller tumors than the control group, which carried shNC-transfected glioma cells (Fig. 3A, B, C). Furthermore, the results of the IHC analysis showed that in mice carrying shMYH9-U87 and shMYH9-LN229 cells, PCNA and MYH9 expression levels were significantly decreased (Fig. 3D). These results suggest that MYH9 expression increases glioma cell proliferation and resistance to TMZ-induced apoptosis.

### MYH9 may increase glioma cell proliferation and resistance to temozolomide via the c-Myc signaling pathway

To identify the mechanism through which MYH9 promotes cell proliferation and resistance to temozolomide, we first used the CPTAC database to analyze proteins related to MYH9 expression. We found that the c-Myc signaling pathway was significantly correlated with MYH9 expression (Supplementary Fig. 1B). As the c-Myc signaling pathway plays a major role in regulating tumor growth [20, 21], we analyzed the relationship between MYH9 expression and the c-Myc signaling pathway. Next, we performed western blotting to identify downstream pathways of MYH9. We found that siMYH9 significantly inhibited the expression of c-Myc (Fig. 3E). In addition, we found that MYH9 knockdown downregulated CCND1 and CDK4 (Fig. 3E). These findings suggested that MYH9 may promote glioma cell proliferation and resistance to temozolomide by regulating the c-Myc pathway.

### MYH9 interacts with NAP1L1 to promote glioma cell proliferation and resistance to temozolomide

**Fig. 4:**
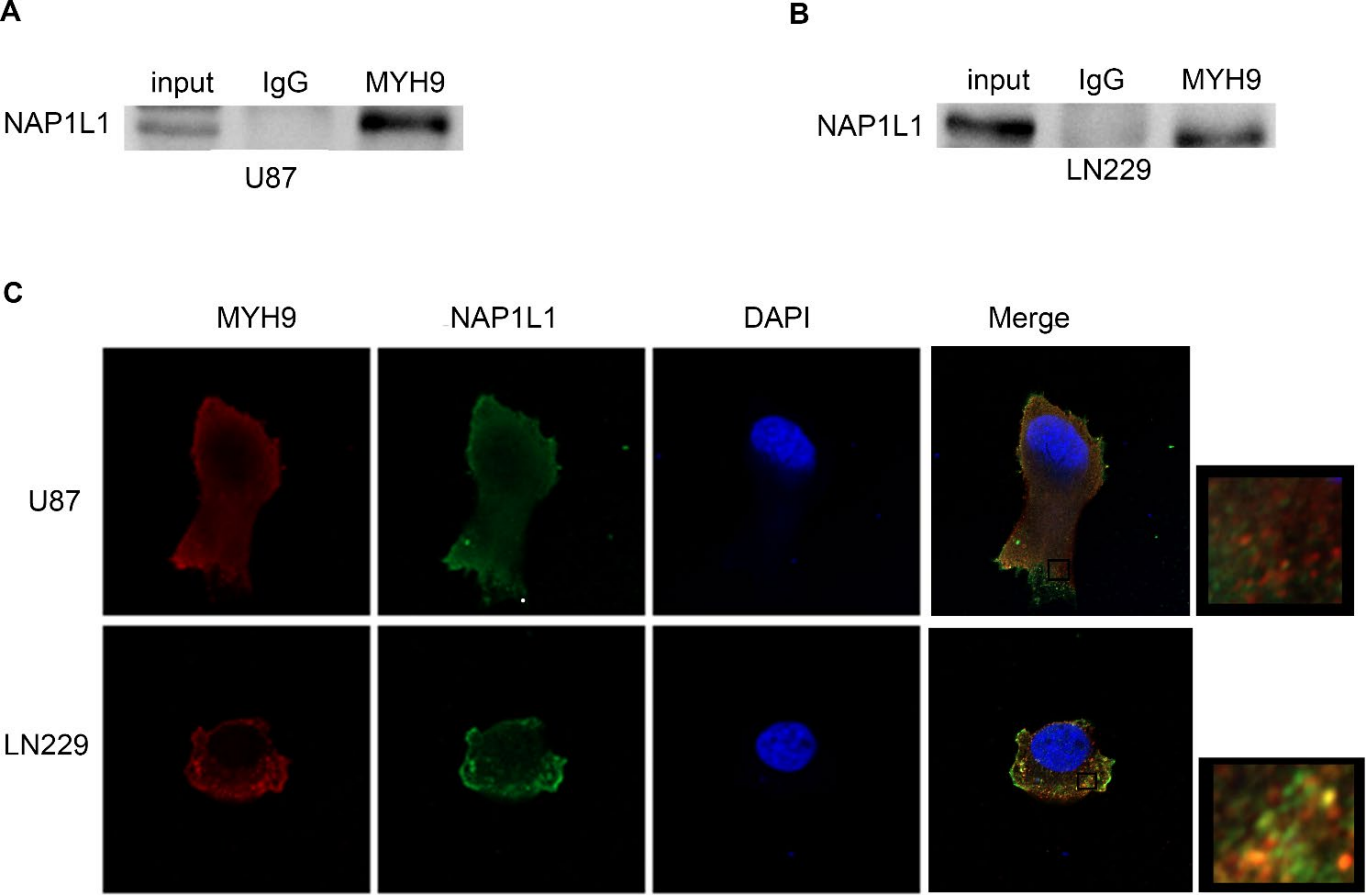
MYH9 interacts with NAP1L1. Co-IP experiments detected the interaction of endogenous MYH9 and NAP1L1 in U87 and LN229 cells. (B) Representative immunofluorescence staining and intensity of MYH9 and NAP1L1 protein in U87 and LN229 cells. Scale bar, 5 μm

The BIOGRID database, combined with Co-IP, was used to explore the molecular mechanisms through which MYH9 regulates the c-Myc pathway in glioma. The interaction between MYH9 and NAP1L1 was investigated using the BIOGRID database. Many studies have found that NAP1L1 is strongly expressed in tumors [22–25], highlighting its potential role in human cancer. A previous study conducted by our group revealed that NAP1L1 enhanced glioma progression by activating the c-Jun signaling pathway. Subsequently, we examined whether MYH9 indeed has relevant binding partners with NAP1L1. The endogenous Co-IP assay showed that MYH9 interacted with NAP1L1 in glioma cells (Fig. 4A, B). Moreover, the immunofluorescence assay revealed that MYH9 and NAP1L1 were primarily present in the cytoplasm of glioma cells (Fig. 4 C). Together, these findings suggest that MYH9 affects glioma progression by interacting with NAP1L1.

### MYH9 inhibited NAP1L1 degradation by stabilizing the USP14-NAP1L1 complex

**Fig. 5:**
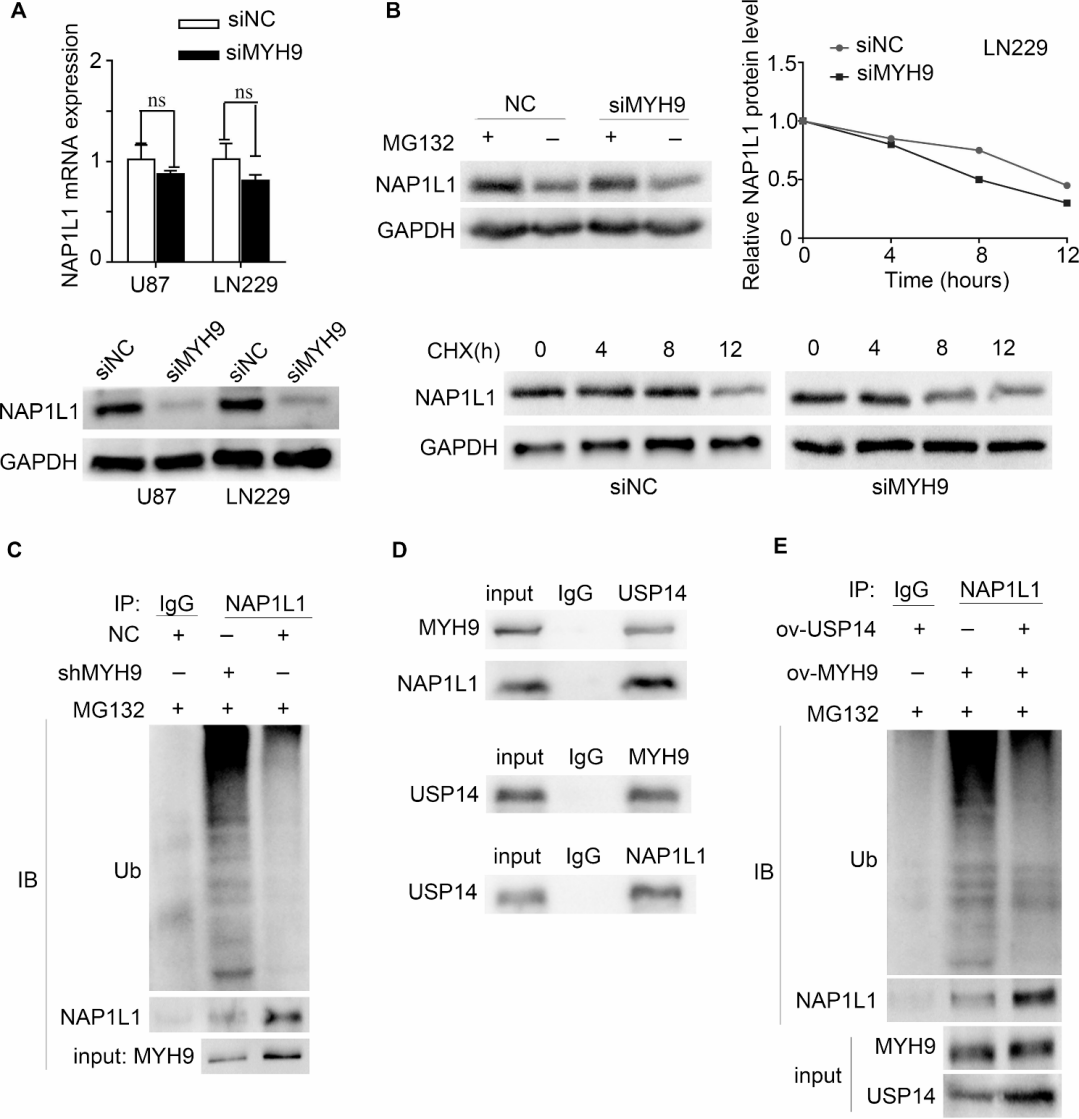
MYH9 inhibits NAP1L1 degradation by stabilizing USP14- NAP1L1 complex. RT-qPCR analysis and WB of NAP1L1 expression in U87 and LN229 cells transfected with siMYH9. (B) WB was used to detect the effects of DMSO or MG132 treatment and CHX treatment for different duration on the stability of NAP1L1 protein in the control and siMYH9 groups. (C) Co-IP detected the effects of MYH9 knockdown on protein stability of NAP1L1 in LN229 cells. (D) Co-IP detected the interaction of MYH9 and NAP1L1 with USP14. (E) Co-IP detected the effects of USP14 overexpression on protein stability of NAP1L1 in MYH9 overexpression in LN229 cells. Data are presented as the mean ± SD for three independent experiments. *P < 0.05, **P < 0.01, ***P < 0.001

In our previous study, we examined an interaction between MYH9 and NAP1L1. To further clarify the role of MYH9 and NAP1L1 in glioma, we attempted to determine whether MYH9 participates in regulating the expression of NAP1L1. Interestingly, our WB results demonstrated that NAP1L1 was markedly downregulated after MYH9 knockdown, while the NAP1L1 mRNA level was unaffected (Fig. 5A). Therefore, it is unlikely that NAP1L1 is regulated by MYH9 at the transcriptional level. Next, whether NAP1L1 is regulated by MYH9 at the posttranscriptional level was evaluated using the cycloheximide (CHX) chasing assay and MG132 treatment. The half-life of NAP1L1 was significantly reduced, and a notable accumulation of NAP1L1 protein was observed in MYH9-silenced cells treated with MG132 and CHX (Fig. 5B). These findings suggested that MYH9 regulates NAP1L1 protein stability. Since proteins can be degraded via the ubiquitin‒proteasome pathway [26], we attempted to determine whether MYH9 regulates NAP1L1 degradation by inhibiting its ubiquitination in glioma cells. Specific anti-NAP1L1 and anti-ubiquitin antibodies were used to perform a co-IP assay. The results of this assay demonstrated that MYH9 greatly decreased the ubiquitination level of NAP1L1 in glioma cells (Fig. 5C).

To study the mechanism through which MYH9 inhibits the degradation of NAP1L1 in more detail, we used the BIOGRID database to predict the interaction between the deubiquitinating enzyme USP14 and NAP1L1. It is interesting to note that no prior studies have shown that USP14 affects the deubiquitination level of NAP1L1. Therefore, we attempted to determine whether USP14 is involved in the MYH9-mediated degradation of NAP1L1. Through endogenous Co-IP, we found that MYH9 and NAP1L1 interacted with USP14 in LN229 and U87 cells (Fig. 5D). In addition, the Co-IP results showed that USP14 silencing enhanced the impact of MYH9 on the ubiquitination and degradation of NAP1L1 (Fig. 5E). These data suggest that MYH9 recruits USP14 to promote NAP1L1 protein stability.

To explore in more detail whether NAP1L1 participates in the promotion of MYH9 in glioma, we transfected MYH9-silenced glioma cells with an NAP1L1 plasmid. The CCK8 assay results showed that NAP1L1 overexpression enhanced cell proliferation in MYH9-silenced glioma cells (Supplementary Fig. 1C). The knockdown of NAP1L1 significantly inhibited the MYH9-mediated activation of the c-Myc pathway and CDK4 and CCND1 expression (Supplementary Fig. 1D). These results indicate that NAP1L1 is involved in MYH9-mediated regulation of the c-Myc pathway in glioma. Altogether, these findings indicate that the MYH9-USP14-NAP1L1 complex plays an important role in glioma progression.

### MYH9 promoted the NAP1L1-cMyc interaction

**Fig. 6:**
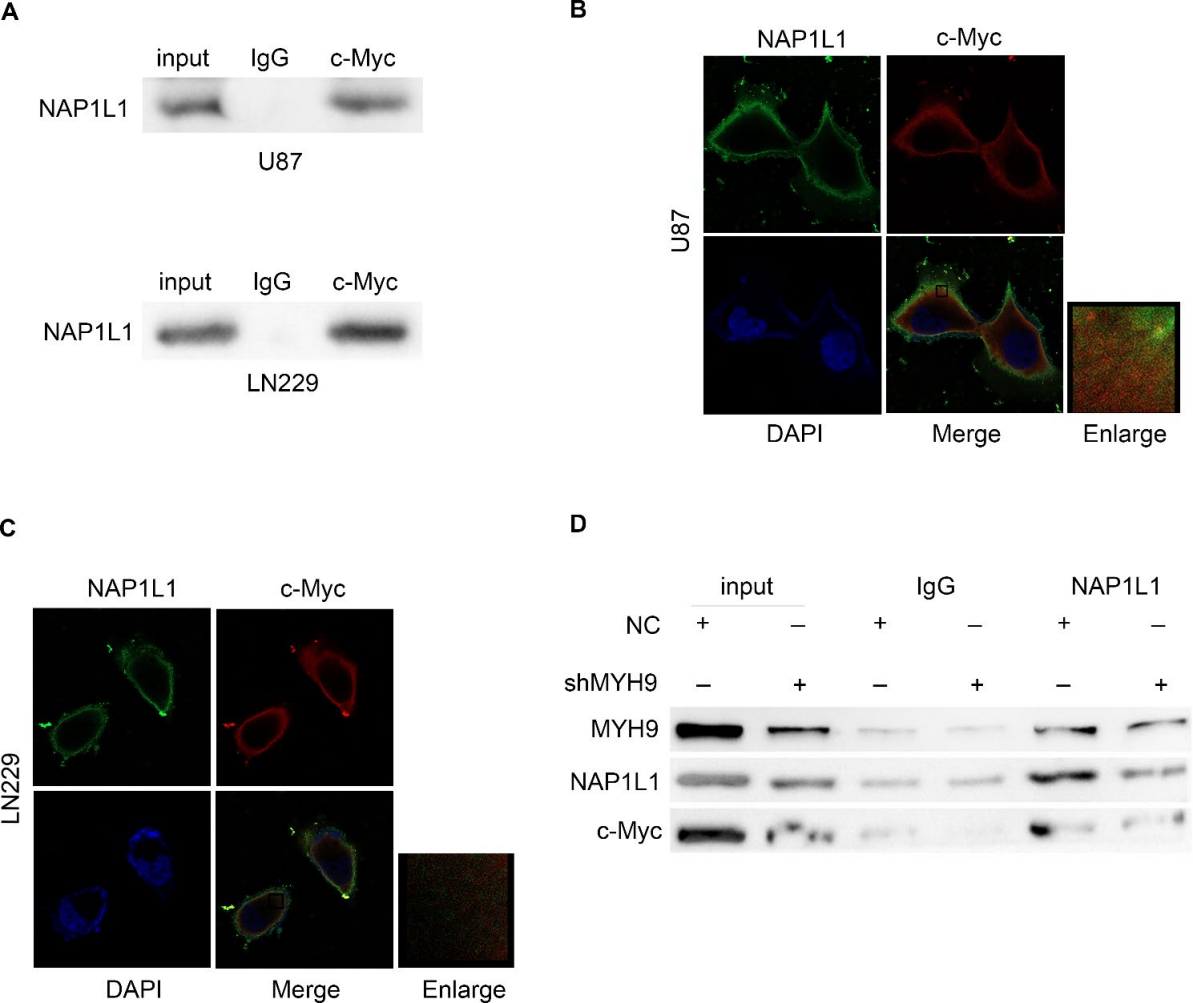
MYH9 inhibits the NAP1L1 -cMyc interaction. (A) Co-IP experiments detected the interaction of endogenous NAP1L1 and c-Myc in U87 and LN229 cells. (B, C) Representative immunofluorescence staining and intensity of NAP1L1 and c-Myc protein in U87 and LN229 cells. Scale bar, 5 μm. (D) Co-IP detected the effects of si-MYH9 on the interaction of NAP1L1 and c-Myc in LN229 cells

In our prior study, we observed that MYH9 can promote c-Myc pathway activation and upregulate the expression of NAP1L1. Therefore, we attempted to determine whether there is a direct relationship between NAP1L1 and c-Myc. We used the BIOGRID database to predict the interaction between c-Myc and NAP1L1. Subsequently, we performed an endogenous Co-IP assay, which showed that NAP1L1 interacted with c-Myc in glioma cells (Fig. 6A). The immunofluorescence assay demonstrated that NAP1L1 and c-Myc mainly colocalized in the glioma cell cytoplasm, with minor nuclear distribution (Fig. 6B, C). Moreover, we found that the interaction between NAP1L1 and c-myc weakened when MYH9 expression levels were reduced (Fig. 6D). These findings suggest for the first time that NAP1L1 activates the c-Myc pathway in glioma cells and that MYH9 promotes the NAP1L1-cMyc interaction.

## MYH9 is an unfavorable factor for glioma

**Fig. 7:**
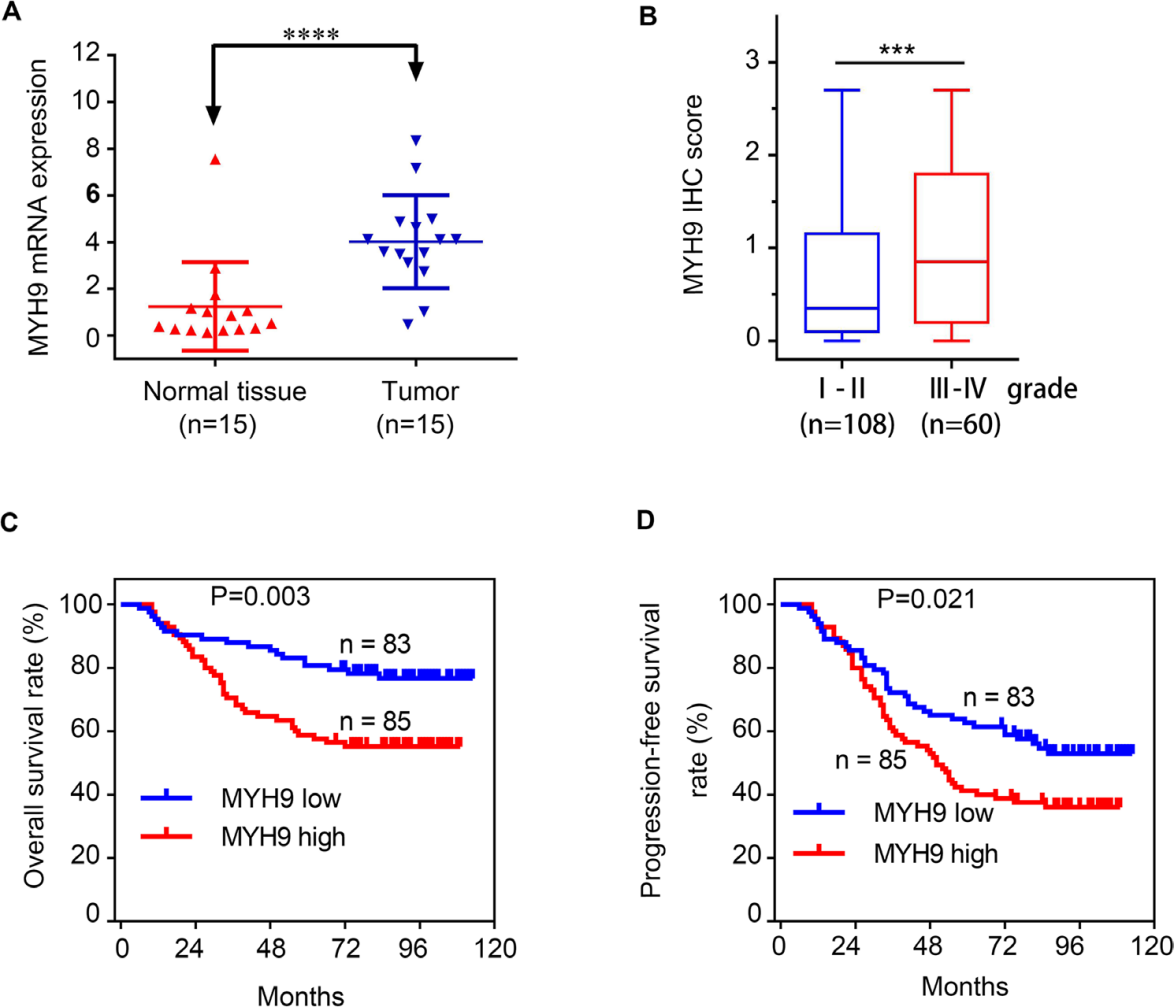
MYH9 as an unfavorable factor in gliomas. RT-qPCR analysis of MYH9 mRNA expression in 15 glioma tissues and 15 para-tumor tissues. (B) IHC score of MYH9 in (gradeI-IV) glioma tissues. (C, D) Kaplan–Meier survival analysis for overall survival (C) and Progression Free survival (D) in TMA showing MYH9 expression. Data are presented as the mean ± SD for three independent experiments. *P < 0.05, **P < 0.01, ***P < 0.001

To verify the functions of MYH9 in glioma cells, we first determined the mRNA levels of MYH9 expression using RT‒qPCR in 15 glioma tissues and 15 paratumoral tissues. The results showed that the MYH9 mRNA expression level was higher in glioma tissues than in paratumoural tissues (Fig. 7A). Using immunohistochemistry (IHC) scores, the MYH9 expression levels in 168 glioma samples were determined (Fig. 7B). Next, we assessed the relationship between glioma patient prognosis and MYH9 expression. We found that elevated MYH9 levels were negatively correlated with the OS and progression-free survival (PFS) of patients with glioma (Fig. 7C, D). It can be seen from Table 1 that the expression of MYH9 in glioma tissues was significantly positively correlated with the Ki-67 index, WHO grades and recurrence. The univariate and multivariate Cox regression analysis results also suggested that MYH9 expression was negatively correlated with the overall survival of glioma patients (Tables 2 and 3).

**Table 1:** Correlation between MYH9 expression and clinicopathological parameters in 168 glioma patients

Characteristics	N	MYH9 expression		*P* value
		Low	High	
Age (years)				
< 60	140	73 (43.4%)	67 (39.9%)	0.112
≥ 60	28	10 (6.0%)	18 (10.7%)	
Gender				
Male	106	51 (30.4%)	55 (32.7%)	0.662
Female	62	32 (19.0%)	30 (17.9%)	
WHO grade				
I-II	108	60 (35.7%)	48 (28.6%)	**0.032**
III-IV	60	23 (13.7%)	37 (22.0%)	
Recurrence				
No	76	45 (26.8%)	31 (18.5%)	**0.021**
Yes	92	38 (22.6%)	54 (32.1%)	
Ki-67 index				
< 20%	87	52(31.0%)	35(20.8%)	**0.005**
≥ 20%	81	31(18.4%)	50(29.8%)	

**Table 2:** Univariate analysis of factors associated with OS and PFS in 168 glioma patients

Factors	OS			PFS
	HR (95% CI)	*P* value	HR (95% CI)	*P* value
**Age (< 60 vs. ≥60)**	2.217 (1.229–3.998)	**0.008**	2.281 (1.409–3.692)	**0.001**
Gender (Male vs. female)	0.669 (0.379–1.181)	0.166	0.719 (0.465–1.113)	0.139
**WHO grade (I-II vs. III-IV)**	10.095 (5.481–18.582)	**0.000**	5.806 (3.784–8.908)	**0.000**
Ki-67 index (< 20% vs. ≥20%)	2.262 (1.312-3.900)	**0.003**	2.061 (1.353–3.137)	**0.001**
**MYH9 expression (Low vs. high)**	2.265 (1.304–3.933)	**0.004**	1.621 (1.069–2.458)	**0.023**

**Table 3:** Multivariate analysis of factors associated with OS and PFS in 168 glioma patients

Factors	OS		PFS	
	HR (95% CI)	*p* value	HR (95% CI)	*p* value
**Age (< 60 vs. ≥60)**	2.141 (1.143–4.012)	**0.017**	2.241 (1.350–3.721)	**0.002**
**WHO grade (I-II vs. III-IV)**	9.723 (5.179–18.253)	**0.000**	5.559 (3.538–8.734)	**0.000**
Ki-67 index (< 20% vs.≥20%)	1.018 (0.563–1.841)	0.954	1.076 (0.675–1.715)	0.757
**MYH9 expression (Low vs. high)**	1.865 (1.065–3.266)	**0.029**	1.203 (0.786–1.842)	0.394

## Discussion

Myosin heavy chain 9 (MYH9), a member of the myosin family of proteins, participates in cell adhesion, polarity, motility, and migration [4]. MYH9 is a skeletal protein that plays a critical role in multiple human diseases [5–9]. Over the past several years, an increasing number of studies have shown that MYH9 is upregulated in various cancers. Nevertheless, the function of MYH9 in glioma has remained unclear. In the present study, using the CPTAC and TCGA datasets, we demonstrated that MYH9 is overexpressed in glioma cells. We used immunohistochemistry to assess MYH9 expression in 168 glioma tissues compared with 15 paratumoral tissues. The expression of MYH9 was positively correlated with the Ki-67 index, recurrence, and WHO grade, indicating that MYH9 contributes to the progression of glioma. Additionally, the upregulated expression of MYH9 was found to be correlated with a poor prognosis for patients with glioma. Next, we found that the knockdown of MYH9 reduced glioma cell proliferation and temozolomide resistance in vitro. Thus, our data indicated that MYH9 is a potential oncogene in glioma.

The BIOGRID database combined with co-IP analysis was used to investigate the molecular mechanisms by which MYH9 promotes the c-Myc pathway in glioma. The BIOGRID database was used to predict the interaction of NAP1L1 with MYH9. NAP1L1 is a family of nucleosome assembly proteins located at 12 q21.2. NAP1L1 can be detected in most tissues and cell lines in humans, but NAP1L1 expression levels are usually higher in rapidly proliferating cells [27, 28]. Many studies have found that NAP1L1 is strongly expressed in tumors [22–25], which has highlighted its potential role in this kind of human disease. A previous study conducted by our group revealed that NAP1L1 enhances glioma progression by activating the c-Jun signaling pathway [29]. Subsequently, we examined whether MYH9 indeed has relevant binding partners with NAP1L1. An endogenous co-IP assay was performed to indicate that MYH9 interacted with NAP1L1 in glioma cells. The immunofluorescence assay demonstrated that MYH9 and NAP1L1 mainly colocalized in the glioma cell cytoplasm. Moreover, the ubiquitination and degradation of NAP1L1 were promoted by MYH9. In this work, we found that the deubiquitination of NAP1L1 was affected by USP14. Importantly, we also found that MYH9 forms a complex with NAP1L1 and USP14, greatly improving their interaction and promoting the USP14-mediated deubiquitination of NAP1L1. In addition, NAP1L1 was found to affect MYH9-mediated regulation of the c-Myc pathway in glioma cells. These findings indicate that MYH9-USP14-NAP1L1 make up a triple complex that is important for the proliferation and temozolomide resistance of glioma cells.

Additionally, to clarify how NAP1L1 promotes the proliferation of glioma cells, we attempted to determine whether there is a direct relationship between NAP1L1 and c-Myc. The BIOGRID database was used to predict the interaction between c-Myc and NAP1L1. Subsequently, the endogenous Co-IP assay indicated that NAP1L1 interacted with c-Myc in glioma cells. Immunofluorescence assay results demonstrated that NAP1L1 and c-Myc mainly colocalized in the glioma cell cytoplasm, with minor nuclear distribution. Furthermore, NAP1L1 silencing severely inhibited the protein expression of c-Myc/CCND1/CDK4. c-Myc is an oncogenic transcription factor in tumors. In prior studies, c-Myc has been shown to regulate CCND1 to induce the cell proliferation of NSCLC [30]. CCND1 is a transcriptional product of c-Myc [31], which is a promoter of the cell cycle leading to cell growth in tumors. [32]. CCND1 forms complexes with CDK4 or CDK6 as a regulator of CDK kinases [33], and its tumor-promoting role has been intensively investigated in human cancers [34]. Altogether, these results demonstrated that c-Myc regulated by NAP1L1 promoted glioma cell proliferation by inducing CCND1/CDK4. Moreover, MYH9 was found to influence the binding between NAP1L1 and c-Myc. These results suggested that MYH9 promotes c-Myc pathway activation via USP14-mediated deubiquitination of NAP1L1, providing a novel mechanism by which MYH9 regulates protein expression related to the proliferation and temozolomide resistance of glioma cells.

**Fig. 8:**
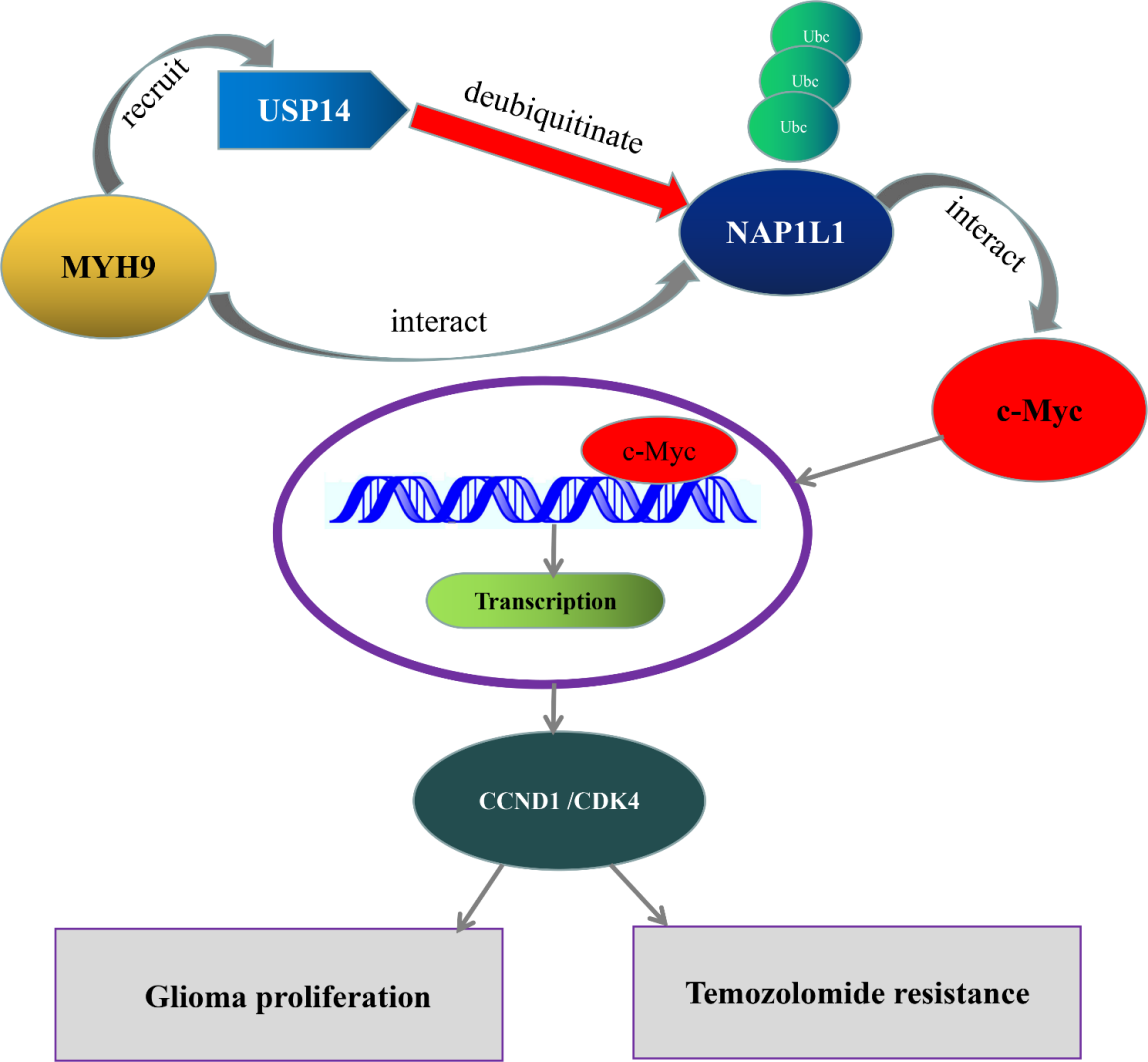
Schematic of MYH9 promoting glioma development. MYH9 bound to NAP1L1, and further inhibited ubiquitination and degradation of NAP1L1 by recruiting USP14. The upregulated NAP1L1 increased the binding with c-Myc and further activated c-Myc, which Induce CCND1/CDK4 expression, and thus promotes the cell proliferation and temozolomide resistance

A regulatory model is shown in Fig. 8 as a summary. MYH9 bound to NAP1L1 to inhibit its ubiquitination and degradation by recruiting USP14. The upregulation of NAP1L1 increased its binding with c-Myc and, further, activated c-Myc, which induced the expression of CCND1/CDK4 and thereby promoted the resistance to temozolomide and proliferation of glioma cells. Higher expression levels of MYH9 indicated shorter survival times in patients with glioma. Overall, these findings suggest that MYH9 has prognostic and therapeutic value for the evaluation and clinical treatment of glioma. The role of MYH9 in glioma should be explored further in future research.

## Materials and methods

### Cells and patients

The U87 and LN229 human glioma cell lines were obtained from the Shanghai Institute of Cell Biology, Chinese Academy of Sciences (Shanghai, China). The cell lines were maintained in DMEM (Gibco) supplemented with 10% fetal bovine serum (FBS, HyClone) at 37 °C and 5% CO_2_. Glioma and paratumoral tissue samples were obtained from the Affiliated Hospital of Southern Medical University. Patients signed an informed consent form before participating in the research. The ethics committee of the Southern Hospital of Southern Medical University approved the research.

### Lentivirus production and infection

Both, a lentivirus carrying the MYH9-shRNA vector (shMYH9) and a lentivirus carrying the negative control vector (shNC), were established by GeneChem (Shanghai, China). Glioma cells was transfected with shMYH9 or shNC, as described in a previous study. The shRNA sequence of MYH9 is presented in Supplementary Table 1. The expression of MYH9 was assessed using qRT-PCR analysis and western blotting.

### Plasmids and small interfering RNAs

USP14 plasmids were obtained from GeneChem (Shanghai, China). siRNAs against MYH9 and NAP1L1 were designed and generated by IGE BIOTECHNOLOGY LTD. (Guangzhou, China). When the cells grew to 70% confluence, the plasmids and siRNAs were transfected into LN229 and U87 cells using lipofactamine ®3000 according to the manufacturer’s instructions (Invitrogen; Thermo Fisher Scientific). The cell culture medium was refreshed after 8 h, and the cells were collected at 36 and 60 h after transfection for use in the next experiment.

### Reverse transcription-quantitative polymerase chain reaction (RT-qPCR)

Cells were lysed and RNA was extracted from them 36 h after transfection using the QIAZOL reagent (Qiagen, Shanghai, China). Complementary DNA (cDNA) was then synthesized from 1 µg extracted RNA using random primers and the Maxima First Strand cDNA Synthesis Kit (Takara Bio, Inc., Otsu, Japan) in the eBio-Rad CFX 96 system. The SYBR®Green Master Mix was used to perform quantitative real-time PCR in the Bio-Rad T100 detection system. All primer sequences are presented in Supplementary Table 2.

### Cell counting kit-8 (CCK8) assay

In order to detect the effect of MYH9 expression on glioma cell viability, the CCK8 assay was used. When cell growth and condition were good, 10% CCK8 reagent was added to each well in a 96-well plate at different time points after siNAP transfection, and the cells were incubated. After 2 h, a Universal Microplate Reader (Bio-Tek instruments, Inc, Winooski, VT, USA) was used to measure cell absorbance (OD) at 450 nm.

### EdU assay

After seeding U87 and LN229 glioma cells in a 96-well plate, their proliferation was examined using a cell-light EDU APOLLO 488 or 567 in vitro imaging kit (Guangzhou Ribobio Co., Ltd). First, we added 50 µM of reagent according to the manufacturer’s instructions. After discarding the waste water, incubate the liquid in the incubation tank for 2 h before washing with PBS. Then, the cells were fixed with 4% polymethylene formaldehyde at room temperature and permeabilized with 0.5% Triton X-100 before being stained for 30 min with a 1x ApolloR staining solution. The F fluid was diluted with deionized water to 1x HOECHEST33342 reaction solution and used to stain cell nuclei for 30 min in a 96-well plate. The number of EDU-positive cells was counted in five random viewing fields using a fluorescence microscope. All tests were performed three times. The Edu assay was carried out in the same manner as described in previous studies.

### Clone formation assay

In order to detect the ability of cells to form clones, we selected 100 cells in good growth condition and plated them in a 6-hole plate per hole. Cross shaking was used to evenly distribute cells. The cells were then incubated for 2 ~ 3 weeks, until clones were visible to the naked eye. Then, the cells were fixed for 10 min with 4% polymethylene and stained with crystalline purple for 30 min. After rinsing off the stain, the number of clones was counted.

### The cycloheximide (CHX) chase assay

First, cycloheximide (Selleck, Cambridge, United Kingdom) was resuspended in DMSO (200 mm) and stored at 20 °C. Then, cells were seeded into a 6-well plate, containing 2 mL of culture medium in each well. Once fixed, the cells were incubated with 50 µg/mL CHX at 37 °C and 5% CO_2_ for various durations. The cells were then collected and analyzed in great detail through western blotting.

### Co-immunoprecipitation (co-IP) assay

After the LN229 and U87 cells reached complete confluence in the culture dishes, 200 µL Pierce IP Lysis buffer (Thermo Scientific) containing protease and phosphatase inhibitor cocktails (Thermo Scientific) in a 100:1:1 proportion was added to the dishes in order to lyse the cells. The cell supernatants were collected 30 min later. The antibodies used in the co-IP assay are presented in Supplementary Table 3. Magnetic beads used for the assay were obtained from Thermo Fisher Scientific. The co-IP assay was conducted according to the manufacturer’s instruction, as previously described [35].

### Western blotting

After 72 h of transfection, cells were washed with a pre-chilled PBS solution three times; then, the were mixed with RIPA lysis buffer (Beyotime Institute of Biotechnology) containing PMSF (Bio-Rad Laboratories, Inc.) and phosphatase inhibitors (Bio-Rad Laboratories, Inc.) (100:1:1) to the cracks for 30 min. The bicinchoninic acid protein assay kit (Beyotime Institute of Biotechnology) was used to detect the total protein content in the cell supernatant, and the upper liquid was further allocated to the ready liquid containing 3 ng/µl protein. Proteins were separated by SDS-PAGE and then transferred onto PVDF membranes (Institute of Biotechnology). The membranes were then blocked with Tween-20 (TBST) in TBS at 37 °C for 1 h. Next, the PVDF membranes were incubated with the appropriate primary antibodies and incubated overnight at 4 °C. Then, the PVDF membranes were incubated with anti-rabbit or anti-mouse IgG secondary antibodies at 37 °C for 1 h. An electrochemiluminescence kit was used to visualize proteins on the PVDF membranes in a dark room, and images of the stained proteins were captured using a ChemiDoc™ Molecular Imager. Each experiment was repeated at least three times.

### Animal studies

Week-old male nude mice purchased from the Experimental Animal Center of Southern Medical University (Guangzhou, China) were used for the animal experiments. The nude mice were placed in a conventional experimental environment, involving alternation between day and night conditions every 12 h and access to sufficient and balanced food and water. Stable LN229 and U87 cells, around 2.0 × 10^6^ in 100 µL PBS, were injected into the bart groin of the nude mice. All nude mice were euthanized after 4 weeks of study. In the euthanasia of nude mice, pentobarbital sodium injection, that is, intraperitoneal injection of the drug 150-200 mg /kg, can stop the animal’s breathing, and if necessary, check whether the animal’s heart is beating. All studies were conducted in accordance with the principles and procedures outlined in the Southern Medical University Guide for the Care and Use of Animals.

### Immunohistochemical (IHC) analysis

Sections of paraffin-embedded mouse tissues obtained for in vivo mouse experiments were used to perform the IHC analysis for evaluating the protein expression levels of MYH9 and PCNA. The indirect streptavidin-peroxidase method was used in accordance with the manufacturer’s recommendations for this analysis, based on a previous study [35]. Supplementary Table 3 lists the antibodies used.

### Statistical analysis

All experimental data is obtained from at least three independent experiments. GraphPad Prism 7.0 (GraphPad Software, Inc., La Jolla, CA, USA) and IBM SPSS 21.0 (IBM Corporation, Armonk, NY, USA) software were used to analysis statistical significant diffference. image processing and calculation of colony intensity were analysed by ImageJ. Data are shown as the means ± SD. Each graph is statistically significant (n s, P > 0.05; *, P < 0.05; **, P < 0.01; ***, P < 0.001). Each trial was performed separately and yielded similar results.

### Electronic supplementary material

Below is the link to the electronic supplementary material.

Supplementary Material 1

Supplementary Material 2

## Data Availability

Data used and/or analyzed in the course of the current study are available from the respective authors upon appropriate request.

## Abbreviations

MYH9: Myosin heavy chain 9
NAP1L1: Nucleosome assembly protein 1 Like 1
TCGA: The cancer genome atlas
IHC: Immunohistochemical
PFS: Progression-free survival
OS: Overall survival
NSCLC: Non-small cell lung cancer
PCNA: Proliferating cell nuclear antigen
CHX: Cycloheximide
RT-qPCR: Real time quantitative polymerase chain reaction
CPTAC: Clinical Proteomic Tumor Analysis Consortium
DFS: Disease Free survival
TMA: Tissue microarray
