# Supplementary figures and images for: Targeting MYH9 represses USP14-mediated NAP1L1 deubiquitination and cell proliferation in glioma
Source: Cancer Cell Int. 2023 Sep 28;23:220. doi: 10.1186/s12935-023-03050-1 (PMC10540345; doi:10.1186/s12935-023-03050-1)

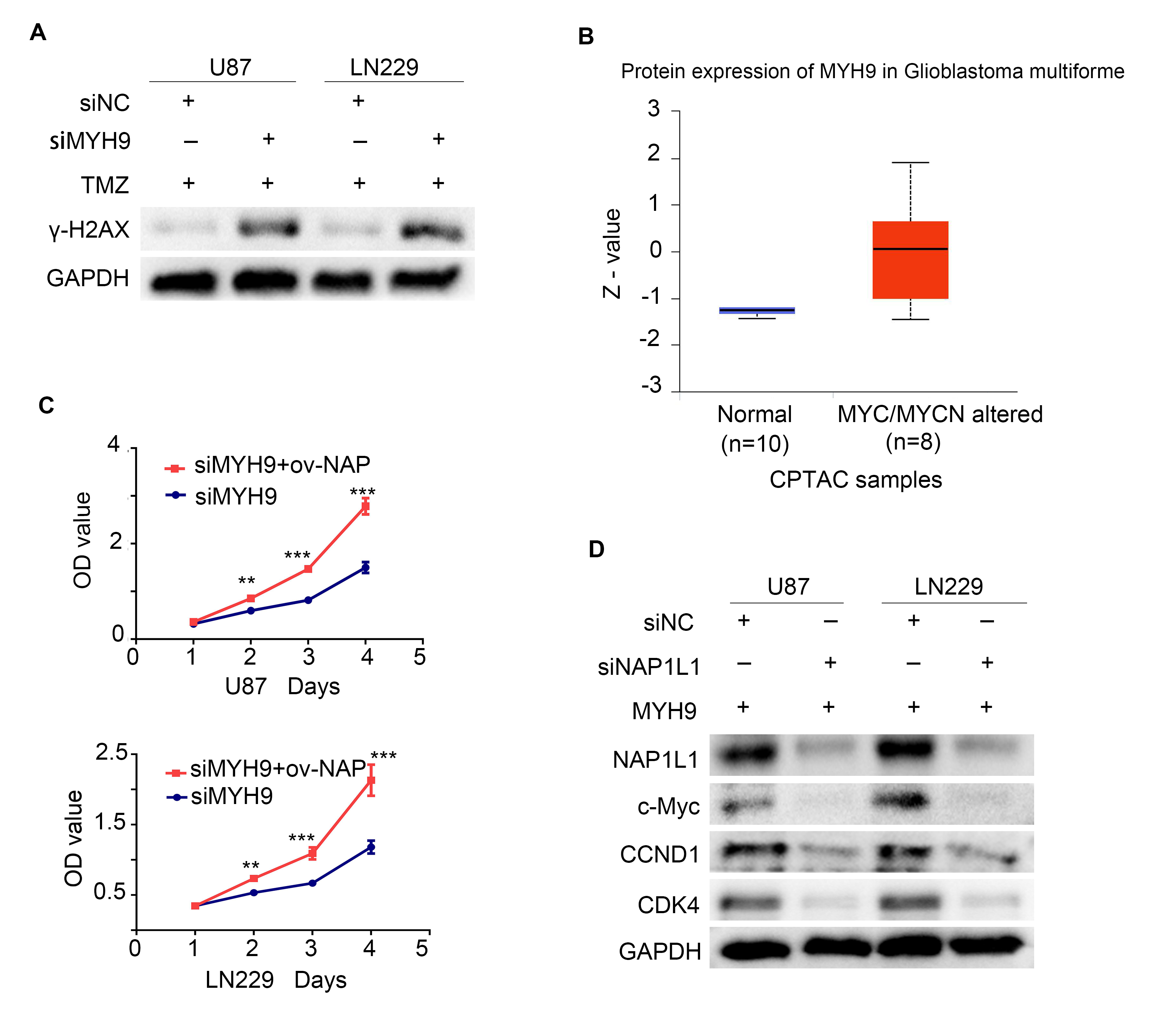

Supplement: Supplementary file 1 — Supplementary Material 1 [file 12935_2023_3050_MOESM1_ESM.tif]
