# Supplementary material for: Targeting MYH9 represses USP14-mediated NAP1L1 deubiquitination and cell proliferation in glioma
Source: Cancer Cell Int. 2023 Sep 28;23:220. doi: 10.1186/s12935-023-03050-1 (PMC10540345; doi:10.1186/s12935-023-03050-1)
Supplement: Supplementary file 2 — Supplementary Material 2 [file 12935_2023_3050_MOESM2_ESM.doc]

**Table S1 The sequences used in this study.**

| **Gene** | **NO** | **Target Sequence** | |
| --- | --- | --- | --- |
| MYH9 | 1 | Sense | 5’ GCAAGCUGCCGAUAAGUAU dTdT 3’ |
|  |  | Antisense | 3’ dTdT CGUUCGACGGCUAUUCAUA 5’ |
|  | 2 | Sense | 5’ GCAAAUUCAUUCGCAUCAA dTdT 3’ |
|  |  | Antisense | 3’ dTdT CGUUUAAGUAAGCGUAGUU 5’ |
| MYH9 shRNA |  | Sense | 5’CGCGTCCCCGCAAACCTCGAGAAGGCAATTCAAGAGATTGCCTTCTCGAGGTTTGCTTTTTGGAAAT 3’ |
|  |  | Antisense | 5’CGATTTCCGCAAACCTCGAGAAGGCAATCTCTTGAATTGCCTTCTCGAGGTTTGCGGGGA 3’ |

**Table S2 The primers used in this study.**

| **Primers name** |  | **Sequence(5’-3’)** |
| --- | --- | --- |
| MYH9 | Forward | AGTTTGTCTCGGAGCTGTGG |
|  | Reverse | GGTTCGTGTTCCTCAGCGTA |
| NAP1L1 | Forward | TTTGCCCCTCCTGAAGTTCC |
|  | Reverse | CCCAACACAACTTGAGACATCC |
| GAPDH | Forward | CATGGGTGTGAACCATGAGA |
|  | Reverse | GTCTTCTGGGTGGCAGTGAT |

**Table S3 A list of Antibodies used for WB, IF, CoIP and IHC.**

| **Antibody** | **Cat.No** | **Company** | **Species** | **Dulution** |
| --- | --- | --- | --- | --- |
| MYH9 | 60233-1-Ig | Proteintech | Mouse | 1:1000(WB); 1:200(IF); 1:20(CoIP) |
| MYH9 | 11128-1-AP | Proteintech | Rabbit | 1:1000(WB); 1:200(IF); 1:300(IHC) |
| USP14 | 14517-1-AP | Proteintech | Rabbit | 1:1000(WB);1:10(CoIP) |
| NAP1L1 | ab178687 | Abcam | Rabbit | 1:1000(WB); 1:200(IF); 1:20(CoIP) |
| NAP1L1 | 14898-1-AP | Proteintech | Rabbit | 1:1000(WB); 1:100(IF); 1:300(IHC) |
| c-Myc | 67447-1-Ig | Proteintech | Mouse | 1:1000(WB); 1:100(IF); 1:10(CoIP) |
| CCND1 | 60186-1-Ig | Proteintech | Mouse | 1:1000(WB) |
| PCNA | 13110 | Cell Sigaling | Rabbit | 1:300(IHC) |
| GAPDH | 10494-1-AP | Proteintech | Rabbit | 1:1000(WB) |
| CDK4 | 11026-1-AP | Proteintech | Rabbit | 1:1000(WB) |
| H2AX | Ab22551 | Abcam | Mouse | 1:1000(WB) |

**Figure legends**

**Fig. S1.** (A) Knockdown of MYH9 promoted the expression of γ-H2AX. (B) The analysis of CPTAC database suggested c-Myc signaling pathway was significantly correlated with MYH9 expression. (C) The CCK8 assay results showed that NAP1L1 overexpression enhanced cell proliferation in MYH9-silenced glioma cells. (D) The knockdown of NAP1L1 significantly inhibited the c-Myc/CCND1/CDK4 expression. Data are presented as the mean ± SD for three independent experiments. *P < 0.05, **P < 0.01, ***P < 0.001.
